# Effects of Combined Extreme Warming and Drought on the Physiology and Growth of *Quercus variabilis* Blume Seedlings

**DOI:** 10.3390/plants15091354

**Published:** 2026-04-28

**Authors:** Se Hee Lee, Ji Won Jang, Seung Hyun Han, Heejae Jo, Gwang-Jung Kim, Yowhan Son, Nam Jin Noh

**Affiliations:** 1 Department of Forestry and Environmental Systems, Kangwon National University, Chuncheon 24341, Republic of Korea; dltpgml0312@kangwon.ac.kr (S.H.L.); beyer100@kangwon.ac.kr (J.W.J.); 2 Forest Technology and Management Research Center, National Institute of Forest Science, Pocheon 11186, Republic of Korea; foresthsh@korea.kr; 3 Department of Environmental Science and Ecological Engineering, Korea University, Seoul 02841, Republic of Korea; avianj@korea.ac.kr (H.J.); yson@korea.ac.kr (Y.S.); 4 Department of Forest Ecology and Management, Swedish University of Agricultural Sciences (SLU), 90183 Umeå, Sweden; gwanjung.kim@slu.se; 5 Department of Forest Resources, Kangwon National University, Chuncheon 24341, Republic of Korea; 6 KNU Convergent Institute for Research on Climate and Long-Term Environmental Change, Kangwon National University, Chuncheon 24341, Republic of Korea

**Keywords:** carbon allocation, climate change, combined stress, oriental oak, open-field experiments, water deficit

## Abstract

Climate change is intensifying extreme climatic events such as warming and drought. This study investigated the physiological and growth responses of *Quercus variabilis*, a major broadleaf plantation species in South Korea, to warming and drought under open-field conditions. From July to August 2024, one-year-old seedlings were exposed to factorial treatments of temperature (ambient: TC; +3 °C: T3; +5 °C: T5) and precipitation (ambient: PC; drought: DR). Gas exchange was measured twice (early: 12 July; late: 16 August) during the treatment period. In the early phase of the experiment, net photosynthetic rate (*P_n_*) was 11.4% lower in DR than in PC, whereas differences were no longer significant in the late phase. Stomatal conductance (*g_s_*) was significantly affected by the interaction between temperature and precipitation. These results suggest that the decline in photosynthesis was driven by non-stomatal limitations such as photosystem II impairment or reduced Rubisco activity, despite maintained or enhanced stomatal conductance. Consequently, intrinsic water-use efficiency (iWUE) during the late phase was 24.3% lower in T5 than in TC. Root collar diameter (RCD) was more sensitive to drought than height growth. Shoot (stem + leaf) biomass was 23.7% higher in T5 than in T3, and root biomass was 20.5% higher in T5 than in TC. However, the root-to-shoot (R/S) ratio did not differ significantly among temperature and precipitation treatments. These findings suggest that *Q. variabilis* seedlings exhibit physiological plasticity and maintain relatively stable biomass allocation under short-term warming and drought conditions.

## 1. Introduction

Climate change is increasing the frequency and intensity of extreme climatic events such as heatwaves and droughts [1,2]. The Intergovernmental Panel on Climate Change (IPCC) reported that the global mean surface temperature during 2011–2020 was 1.1 °C higher than during 1850–1900 and projected a further increase of 1.4–4.4 °C by 2081–2100 if current warming trends persist [3]. In parallel, droughts are becoming more severe and prolonged, with an increasing likelihood of developing into multiyear events [4]. These trends are destabilizing climatic regimes and disrupting seasonal patterns, thereby increasing environmental variability [5]. Accordingly, understanding the physiological and growth responses, as well as the adaptive strategies, of tree seedlings under extreme climatic events, has become increasingly important.

Extreme warming and drought strongly influence plant water relations, stomatal regulation, photosynthesis, and growth [6,7]. Moderate warming can enhance photosynthetic performance in seedlings and promote growth. Under high-temperature stress, however, this limitation shifts toward non-stomatal limitation, associated with biochemical impairments such as reduced photosystem II activity and decreased efficiency of carbon assimilation enzymes, ultimately suppressing photosynthetic processes [8,9,10]. Drought reduces water availability for trees, leading to decreased water-use efficiency, reduced cellular turgor, and constrained height and root collar diameter growth [11]. In response to water stress, plants generally adopt either isohydric or anisohydric strategies; isohydric species typically stabilize leaf water potential by reducing stomatal conductance, whereas anisohydric species often prioritize carbon assimilation by maintaining stomatal opening, even at the cost of a significant decline in leaf water potential [12,13,14]. Although many tree species exhibit partial tolerance to thermal or water stress [15], extreme conditions can delay recovery or cause irreversible damage. When warming and drought occur simultaneously, their effects often interact, disrupting water-use strategies and carbon metabolism, ultimately limiting height and diameter growth as well as biomass accumulation [16,17].

Under concurrent warming and drought, plants face a critical trade-off between stomatal closure for water conservation and the maintenance of transpirational cooling to mitigate thermal damage [18,19]. Notably, previous studies have reported a decoupling between stomatal conductance and photosynthesis under extreme warming; transpiration often persists even when carbon assimilation is severely inhibited [20,21,22,23]. This phenomenon has been partly attributed to the temperature-induced reduction in water viscosity, which facilitates hydraulic transport to evaporative sites within the leaf mesophyll [24]. Nevertheless, the precise physiological mechanisms driving this decoupling remain to be fully elucidated [24]. These functional imbalances are closely linked to allometric shifts in biomass allocation [25,26,27,28]. Plants typically prioritize root development over shoot growth to enhance water uptake under deficit conditions [29]. Seedlings are particularly vulnerable due to their shallow root systems and limited internal reserves [30,31], as repeated stress exposure may fundamentally restructure their carbon allocation strategies.

In this study, we focused on *Quercus variabilis* Blume, a dominant broad-leaved species in South Korea. Oak forests, comprising various *Quercus* species, constitute approximately 16.5% of the national forest area [32]. Among these, *Q. variabilis* is recognized for its resilience to drought and fire, as well as its significant capacity for carbon sequestration. Previous studies have demonstrated that oaks can facilitate ecosystem resilience under recurrent droughts and summer conditions [33,34,35]. However, the physiological and growth response mechanisms of *Q. variabilis* seedlings under combined warming and drought stress remain poorly understood. Consequently, we conducted an open-field simulation of extreme summer conditions to evaluate the photosynthetic and growth responses of *Q. variabilis* seedlings to combined warming and drought. We hypothesized that:

**H1.**  *Combined extreme warming and drought would induce stomatal closure, thus suppressing transpiration and net photosynthetic rates.*

**H2.**  *Water deficit would promote belowground biomass allocation to enhance water acquisition, increasing the root-to-shoot ratio.*

## 2. Results

### 2.1. Environmental Factors

**Figure 1:**
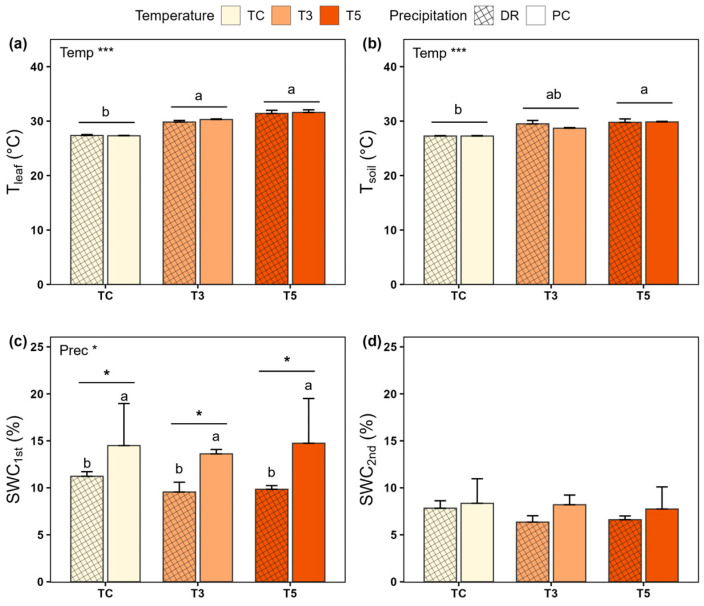
Changes in environmental factors under extreme warming and drought conditions. (**a**) Leaf temperature (T_leaf_) during the entire temperature treatment period; (**b**) soil temperature (T_soil_) during the entire temperature treatment period; (**c**) soil water content (SWC) during the P1st precipitation treatment period; (**d**) SWC during the P2nd precipitation treatment period. TC: ambient temperature; T3: +3 °C warming; T5: +5 °C warming; PC: ambient precipitation; DR: drought condition. Asterisks indicate significant effects of factors or their interaction (* *p* < 0.05; *** *p* < 0.001). Different letters indicate significant differences among temperature treatments (T_leaf_ and T_soil_) and between precipitation treatments within each temperature (SWC1st).

During the experimental period (July–August), temperature and precipitation treatments were applied three and two times, respectively. Target leaf temperature (T_leaf_) increases were set at 3 °C (T3) and 5 °C (T5) relative to TC, with actual increases closely matching these at 2.6–2.8 °C and 4.4–4.5 °C, respectively. Warming significantly increased both T_leaf_ (°C) and soil temperature (T_soil_, °C) throughout the entire treatment period (Table 1; Figure 1a,b). Across all plots, T_leaf_ (mean ± SE) is higher in T5 (31.08 ± 0.67) and T3 (30.15 ± 0.30) than in TC (27.57 ± 0.80). Similarly, T_soil_ is higher in T5 (29.74 ± 0.57) than in TC (27.19 ± 0.50).

When examining each period separately (Figure S1a,b), T_leaf_ and T_soil_ were consistently higher in T3 and T5 than in TC. For T_leaf_, the order was T5 > T3 > TC in both T1st (29.59 ± 0.06, 27.85 ± 0.02, and 25.04 ± 0.02) and T3rd (33.61 ± 0.10, 32.00 ± 0.08, and 29.20 ± 0.05). In T2nd, T5 (31.08 ± 0.16) and T3 (30.15 ± 0.07) were comparable, and both exceeded TC (27.57 ± 0.02). T_soil_ showed similar trends: T5 > T3 > TC in T1st (27.19 ± 0.05, 26.25 ± 0.05, and 24.69 ± 0.01). In T2nd and T3rd, T5 (29.42 ± 0.10, 32.62 ± 0.08) and T3 (28.90 ± 0.09, 31.90 ± 0.09) showed no significant differences, although both were significantly higher than TC (27.13 ± 0.02, 29.75 ± 0.02). Soil water content (SWC) was lower in DR (10.2 ± 0.09%) than in PC (14.3 ± 0.39%) during P1st (Figure 1c and Figure S1c). Although no overall differences among treatments were observed during the P2nd period, significant reductions in SWC were observed on specific dates (Figure 1d and Figure S1c), with DR (8.19 ± 0.30%) being significantly lower than PC (10.50 ± 0.71%).

### 2.2. Gas Exchanges and iWUE

**Figure 2:**
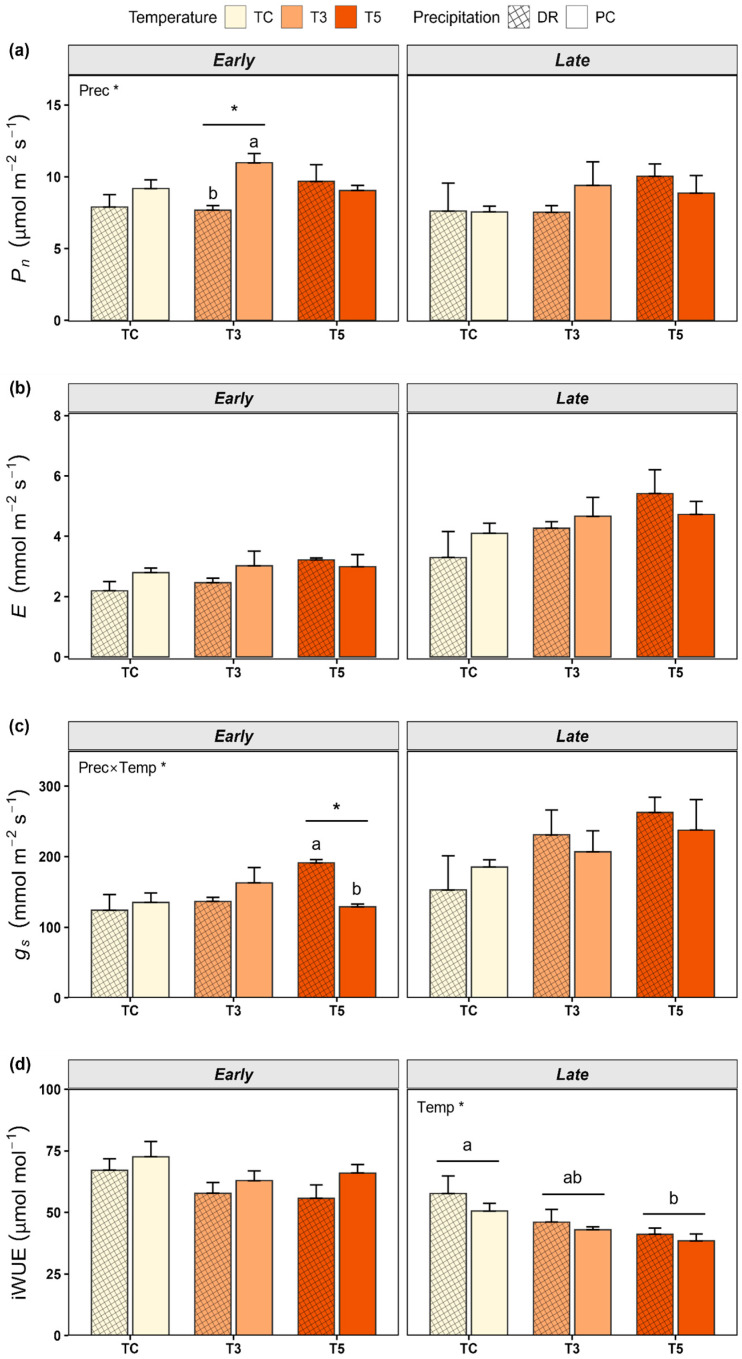
Changes in gas exchange parameters and intrinsic water-use efficiency (iWUE) under extreme warming and drought conditions. (**a**) Net photosynthetic rate (*P_n_*); (**b**) transpiration rate (*E*); (**c**) stomatal conductance (*g_s_*); (**d**) iWUE. Early and late phases indicate measurement time points during the treatment period (12 July and 16 August, respectively). TC: ambient temperature; T3: +3 °C warming; T5: +5 °C warming; PC: ambient precipitation; DR: drought condition. Asterisks indicate significant effects of factors or their interaction (* *p* < 0.05). Different letters indicate significant differences between precipitation treatments within each temperature (*P_n_* and *g_s_*) and among temperature treatments (iWUE).

In the early phase, net photosynthetic rate *(P_n_)* differed significantly between precipitation treatments (Table 2; Figure 2), being lower in DR (8.42 ± 0.53 μmol m^−2^ s^−1^) than in PC (9.73 ± 0.41 μmol m^−2^ s^−1^). This difference was more pronounced under T3, where *P_n_* was lower in DR (7.67 ± 0.32 μmol m^−2^ s^−1^) than in PC (10.98 ± 0.64 μmol m^−2^ s^−1^). A significant interaction between temperature and precipitation was observed for stomatal conductance (*g_s_*). Under T5, *g_s_* significantly increased in DR (191.12 ± 4.74 mmol m^−2^ s^−1^) compared to PC (128.74 ± 4.10 mmol m^−2^ s^−1^), whereas no significant differences between precipitation treatments were observed under TC or T3. Transpiration rate (*E*) and intrinsic water-use efficiency (iWUE) showed no significant differences in response to temperature or precipitation, or their interaction.

In contrast to the early phase, *P_n_*, *E*, and *g_s_* did not differ significantly in response to temperature, precipitation, or their interaction in the late phase (Figure 2). Both *E* and *g_s_* tended to increase under warming treatments (T3 and T5) relative to TC, regardless of precipitation conditions. In contrast, iWUE was significantly affected by temperature, with lower values in T5 (39.80 ± 1.77 μmol mol^−1^) than in TC (54.13 ± 3.81 μmol mol^−1^).

**Figure 3:**
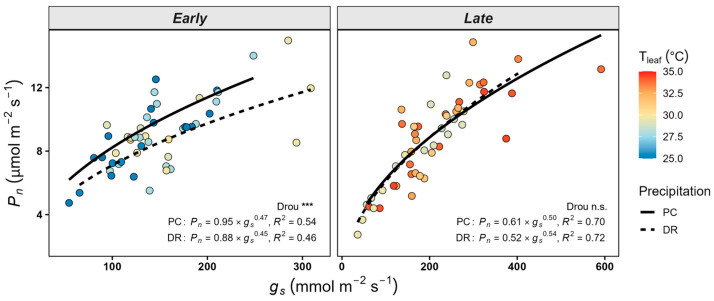
Relationship between net photosynthesis rate (*P_n_*) and stomatal conductance (*g_s_*). Early and late phases indicate measurement time points during the treatment period (12 July and 16 August, respectively). PC: ambient precipitation; DR: drought condition. Point color indicates leaf temperature (T_leaf_), and solid and dashed lines represent PC and DR, respectively. Asterisks indicate significant differences between PC and DR within each phase (*** *p* < 0.001; n.s., non-significance).

Regardless of measurement timing and precipitation treatment, *P_n_* increased with increasing stomatal conductance *g_s_* (Figure 3). In the early phase, the relationship between *P_n_* and *g_s_* differed between precipitation treatments (PC: R^2^ = 0.54; DR: R^2^ = 0.46). However, in the late phase, no significant difference was observed between treatments (PC: R^2^ = 0.70; DR: R^2^ = 0.72).

### 2.3. Growth and Biomass

**Figure 4:**
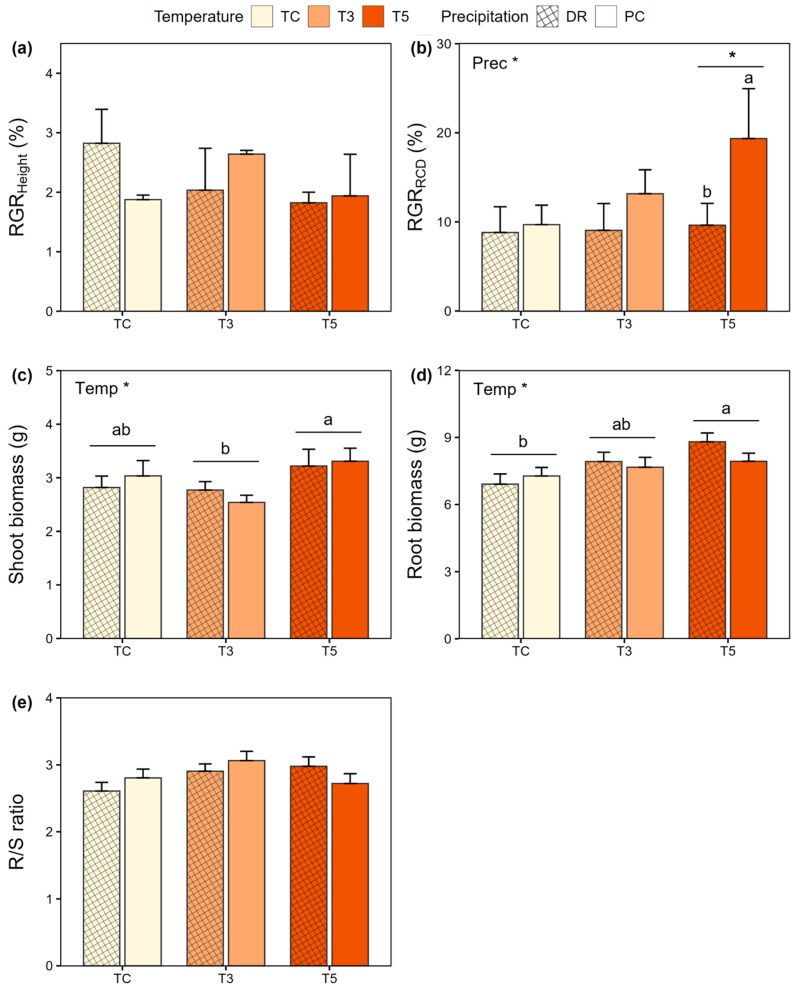
Changes in growth and biomass under extreme warming and drought conditions. (**a**) Relative growth rate (RGR) of height; (**b**) RGR of root collar diameter (RCD); (**c**) shoot biomass; (**d**) root biomass; (**e**) root-to-shoot (R/S) ratio. TC: ambient temperature; T3: +3 °C warming; T5: +5 °C warming; PC: ambient precipitation; DR: drought condition. Asterisks denote statistically significant differences (* *p* < 0.05). Different letters indicate significant differences between precipitation treatments within each temperature (RGR_RCD_) and among temperature treatments (shoot and root biomass).

The root collar diameter (RCD) growth of *Q. variabilis* was more sensitive to simulated extreme climate conditions than height growth, with relative growth rate (RGR) and absolute growth rate (AGR) showing consistent patterns (Table 2 and Table S1). RGR_RCD_ was significantly affected by precipitation treatments, being lower in DR (9.63 ± 2.45%) than in PC (19.36 ± 5.60%) at T5, whereas no significant differences were observed under TC or T3 (Figure 4b). AGR_RCD_ showed a similar pattern, with lower values in DR (0.28 ± 0.07 mm) than in PC (0.54 ± 0.14 mm) at T5 (Figure S2b). In contrast, neither RGR_Height_ nor AGR_Height_ was significantly affected by temperature, precipitation treatment, or their interaction (Figure 4a and Figure S2a).

Shoot (stem + leaf) and root biomass were significantly affected by temperature, whereas stem biomass showed no significant response to temperature, precipitation, or their interaction (Table 2). Shoot biomass was highest in T5 (3.28 ± 0.21 g) and lowest in T3 (2.66 ± 0.08 g) (Figure 4c). Similarly, leaf biomass was highest in T5 (2.22 ± 0.15 g) and lowest in T3 (1.79 ± 0.06 g) (Figure S3). Root biomass was significantly greater in T5 (8.38 ± 0.28 g) than in TC (7.04 ± 0.34 g) (Figure 4d). However, the root-to-shoot (R/S) ratio did not differ significantly among treatments (Figure 4e).

## 3. Discussion

### 3.1. Changes in Environmental Factors

The warming manipulation increased T_leaf_ to levels comparable to the target warming levels and also elevated T_soil_, confirming the effectiveness of the warming treatment. The precipitation manipulation had a significant effect on SWC1st, whereas no significant differences were observed in SWC2nd. This pattern was likely due to negligible rainfall (0.5 mm) during 1–4 August of the second precipitation manipulation, which prevented clear differences between the precipitation treatments from emerging. However, soil water content differed between PC and DR during rainfall events (Figure S1c). A substantial rainfall event (11.5 mm) on 5 August increased soil moisture in PC relative to DR, making the treatment differences apparent [36].

### 3.2. Changes in Gas Exchanges

In the early phase, precipitation manipulation significantly reduced *P*_n_ by 11.4% under DR compared with PC (Figure 2). In contrast, stomatal conductance exhibited a significant interaction between temperature and precipitation, with higher *g_s_* under T5DR, whereas no significant differences in *E* were observed among treatments (Figure 2). Generally, stomatal conductance and photosynthesis are closely coordinated, and photosynthetic decline under water deficit is often attributed to stomatal limitation [37,38,39,40]. Xiong et al. (2022) reported that seedlings of *Q*. *fabri*, *Q. serrata*, *Q. acutissima*, and *Q. variabilis* showed substantial reductions in photosynthesis, transpiration, and stomatal conductance under prolonged drought, with photosynthesis decreasing by more than 60% after 31 days [41]. However, our study showed a decline in photosynthesis despite maintained or even increased stomatal conductance under certain conditions. This pattern suggests that the decline in photosynthesis was not explained by stomatal limitation alone and was likely associated with non-stomatal processes, possibly reflecting the combined influence of multiple physiological constraints [42]. Mwangi et al. (2025) reported that under severe drought conditions—defined as Standardized Precipitation Index (SPI) ≤ −2.0 or soil water content below 0.15 m^3^ m^−3^—non-stomatal limitations, primarily photodamage to photosystem II and Rubisco inactivation, may become the dominant constraints on photosynthesis [43]. Also, Chaves et al. (2008) reported that drought-induced declines in photosynthesis can also arise from non-stomatal limitations, including reductions in mesophyll conductance and impairments in biochemical and photochemical processes [44].

Moreover, in our study, stomatal conductance under T5DR was higher than under T5PC (Figure 2). These results suggest that water limitation did not strongly reduce stomatal conductance at high temperatures. Gauthey et al. (2024) found that in mature *Q. ilex* and *Q. coccifera*, stomatal conductance remained low but positive even when net photosynthesis approached zero under maximum air temperatures of up to 42 °C [45]. This stomatal response may be attributed to multiple interacting physiological mechanisms. Elevated temperatures can enhance guard cell activity, promoting stomatal opening [46]. Moreover, as stomatal behavior is regulated by the integration of multiple environmental signals rather than by abscisic acid alone [47], signals promoting stomatal opening may have contributed to the observed patterns under warming conditions. This maintained stomatal opening under combined stress may be associated with sustained transpiration under warming conditions. Marchin et al. (2022) demonstrated, in a glasshouse experiment with seedlings of 20 broadleaf species, that droughted plants can contribute more strongly to transpirational leaf cooling during heatwaves, maintaining stomatal opening despite water limitation [48]. Likewise, Drake et al. (2018) reported that *Eucalyptus parramattensis* seedlings exposed to a four-day heatwave (>43 °C) following one month of drought exhibited nearly zero photosynthesis, whereas transpiration persisted to sustain canopy cooling [22]. Additionally, stomatal conductance exhibited a significant interaction between temperature and precipitation, whereas photosynthesis did not, which may reflect both the relatively short treatment duration and a temporal shift in the dominant limiting factors. This response may also be associated with anisohydric-like stomatal behavior in *Q. variabilis*, characterized by the maintenance of stomatal conductance under stress and a potential decoupling between photosynthesis and stomatal conductance. Therefore, these results suggest that *Q. variabilis* responds sensitively to extreme drought conditions during the early phase through reductions in photosynthesis, while maintaining transpiration, which may contribute to leaf temperature regulation. Hypothesis **H1** was not supported. This pattern of reduced photosynthesis despite maintained stomatal conductance further supports a decoupling between photosynthesis and stomatal conductance under warming and drought (Figure 3 and Figure S4). However, as gas exchange measurements were conducted on a single leaf per seedling, the sample size at the leaf level was limited, and thus caution is warranted in interpreting the results, particularly with respect to within-plant variability.

In the early phase, *P_n_* was primarily regulated by water availability; however, during the late phase, differences between precipitation treatments were no longer observed as the treatments were prolonged and repeatedly imposed (Figure 3). This transition suggests that the dominant environmental constraint may have shifted from water deficit to heat stress as the experiment progressed, with sustained high-temperature stress potentially overriding the influence of water availability during the late phase [26,49]. This interpretation may be limited by the short-term nature of the experiment and may not reflect longer-term acclimation processes. Teskey et al. (2015) demonstrated that heat stress can induce not only immediate damage but also persistent and lagged physiological effects through cumulative stress processes [8]. Consistent with this, Ruehr et al. (2019) reported that recovery of gas exchange can be delayed, potentially due to impaired photosynthetic function and persistent leaf damage at temperatures exceeding 40 °C [15]. Alternatively, the lack of treatment effects on *P_n_* may also reflect relatively similar soil water content levels between precipitation treatments during the second precipitation treatment period.

In our study, iWUE declined significantly under warming during the late phase, whereas *g_s_* and *E* remained at similar or slightly higher levels compared to the early phase, without significant differences among treatments. Notably, this decline in iWUE occurred despite the lack of treatment effects on *P_n_*, suggesting a potential decoupling between carbon gain and water loss. This pattern suggests that seedlings may have maintained transpiration under warming, potentially contributing to leaf temperature regulation. However, photosynthetic recovery likely remained constrained by non-stomatal limitations, leading to reduced water-use efficiency [18,22,50]. Consequently, these results indicate that *Q. variabilis* seedlings initially exhibited physiological plasticity that allowed rapid acclimation to environmental stress. Over time, however, the physiological response shifted toward maintaining thermal stability under sustained warming and drought conditions, which resulted in a decrease in carbon assimilation efficiency.

### 3.3. Changes in Growth and Biomass

Warming is known to promote plant growth [51,52]; however, such positive effects are constrained under extreme drought conditions [53]. In our study, the RCD of *Q. variabilis* seedlings exhibited greater sensitivity to extreme climate conditions than height growth, with a particularly pronounced reduction observed under DR (Figure 4 and Figure S2). Overall, these results indicate that water deficit constrained plant growth by reducing photosynthetic carbon assimilation and subsequent carbon availability [54,55]. This response is consistent with previous findings for *Larix kaempferi* [56] and *Q. variabilis* [57], but contrasts with patterns reported for *Abies koreana* [58] and *L*. *kaempferi* [59]. This suggests that species-specific water regulation strategies and differences in stress intensity play important roles in shaping growth responses.

The observed growth responses may be related to anisohydric-like stomatal behavior in *Q. variabilis*. In our study, elevated stomatal conductance during the early phase of combined extreme warming and drought (T5DR), relative to the precipitation control, may be associated with reduced RCD growth observed under these stress conditions. Although stomatal opening is advantageous for regulating leaf temperature under warming [24], it can reduce plant water potential and limit the maintenance of turgor pressure required for cell expansion and radial growth [60,61]. Consequently, RCD growth—which is particularly sensitive to water stress—declined under drought conditions, whereas height growth remained largely unaffected. This pattern suggests a physiological trade-off in which growth was preferentially maintained in height rather than stem thickening. Leaf and root biomass were significantly influenced by temperature, with the highest values under warming treatments. Although anisohydric species are susceptible to excessive water loss and leaf shedding [62], they often maintain relatively high stomatal conductance under combined drought and warming, thereby allowing continued carbon assimilation. In addition, enhanced root growth under elevated evaporative demand may represent a short-term physiological adjustment that improves water uptake capacity in *Q. variabilis* seedlings during extreme climatic events [63].

In contrast, the R/S ratio did not differ significantly among treatments, which differs from previous studies reporting increased belowground allocation under warming and drought. For example, Taeger et al. (2015) showed that simulated drought (6 months) increased taproot length and the R/S ratio in *Pinus sylvestris* seedlings [64]. Arend et al. (2011) reported that the root length-to-shoot height ratio in two-year-old seedlings of *Q. robur*, *Q. petraea*, and *Q. pubescens* increased under drought but declined under warming conditions over three years of growing seasons [65]. In contrast to *Q. robur*, which is relatively sensitive to soil water depletion and tends to increase root length to enhance water uptake, *Q. variabilis* is considered a relatively drought-tolerant species [41,65]. Such tolerance may allow *Q. variabilis* to maintain relatively stable biomass allocation patterns rather than undergoing pronounced structural adjustments, particularly under short-term and combined warming and drought stress. Furthermore, the stable R/S ratio may reflect a prioritization of internal carbon storage over structural adjustments. Kannenberg et al. (2017) suggested that coarse roots can buffer whole-tree carbon pools by shifting allocation toward non-structural carbohydrates during stress, even when growth is suppressed [66]. This indicates a trade-off between structural expansion and storage, enabling *Q. variabilis* to maintain biomass balance under acute stress conditions [66]. Overall, Hypothesis **H2** was rejected, as root biomass responded more sensitively to temperature than to water availability, and the R/S ratio remained unaffected by both warming and soil water conditions. These findings indicate that, in contrast to the typical drought-avoidance strategy characterized by elevated R/S ratios, *Q. variabilis* seedlings maintained relatively stable biomass allocation patterns even under short-term extreme climatic treatments, while drought sensitivity was expressed primarily through reduced RCD growth. These conservative allocation responses may reflect the restriction of the treatment period to summer and species-specific acclimation responses.

## 4. Materials and Methods

### 4.1. Study Site

**Figure 5:**
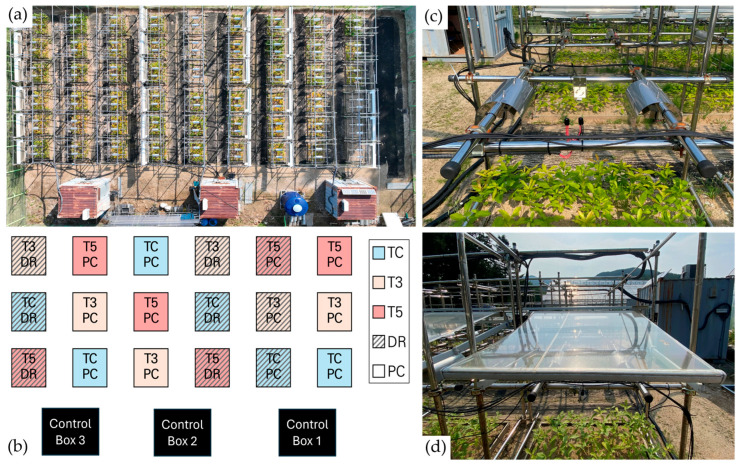
Experimental setup for open-field extreme climate manipulation. (**a**) Overview of the experimental site; (**b**) schematic diagram of the experimental design showing temperature and precipitation treatments; (**c**) warming manipulation system using infrared heating lamps; (**d**) drought manipulation system using automated rain shelters.

The open-field experiment was conducted in 2024 at the Forest Technology and Management Research Center (37°45′39″ N, 127°10′15″ E; 106 m a.s.l.) in Pocheon, Gyeonggi-do, South Korea (Figure 5). The field has a temperate climate with hot, humid summers and cold winters. The mean annual temperature and total annual precipitation (1908–2023) were 11.8 °C and 1320.9 mm yr^−1^, respectively [36]. The mean temperatures for July and August 2024 were 26.6 °C and 29.3 °C, with total monthly precipitation of 557.3 mm and 72.8 mm, respectively. The soil properties of the study site were as follows: loamy sand (sand 86.7%, silt 10.6%, clay 2.8%), pH 6.31 ± 0.12, electrical conductivity 0.18 ± 0.02 dS cm^−1^, total carbon concentration 0.86 ± 0.08%, and total nitrogen concentration 0.05 ± 0.01%.

### 4.2. Experimental Design

The experiment was arranged in three blocks, each containing six plots (1.5 × 1.0 m). Treatments consisted of a factorial combination of two factors: temperature and precipitation. Temperature treatments were ambient (TC), ambient +3 °C (T3), and ambient +5 °C (T5), whereas precipitation treatments were ambient (PC) and drought conditions (DR). Thus, a total of 18 plots were established (3 temperature levels × 2 precipitation levels × 3 replicates). One-year-old *Q*. *variabilis* seedlings were planted in May 2024, with 63 seedlings (7 × 9) per plot [67]. To minimize variation in seedling size among plots, seedlings of different sizes were evenly distributed.

The leaf temperature (T_leaf_) of all plots was measured using an infrared thermometer (SI-111-SS, Apogee Instruments, Logan, UT, USA). A relay controller (SDM-CD-16AC, Campbell Scientific Instruments, Logan, UT, USA) operated an infrared heater (FT-1000, Mor Electronic Heating Associates Instruments, Comstock Park, MI, USA) to maintain the target temperature. The heaters automatically turned on when the temperature in the T3 and T5 plots fell below their target levels (3 °C and 5 °C above TC, respectively) and turned off once the target was reached. In the TC plots, a cover for the infrared heater was installed without the heater to replicate the shading effects present in the other temperature treatments. A rain detector (BSR-307, KJ Green Systems Co., Ltd., Busan, Republic of Korea) automatically deployed a transparent rainout shelter (2.0 × 1.5 m) during rainfall. To prevent errors caused by frequent activation, the rainout shelter remained operational for 30 min even when no rain was detected. Soil water content (SWC) and temperature sensors (CS655, Campbell Scientific Instruments, Logan, UT, USA) were installed at a depth of 10–15 cm to monitor soil conditions. T_leaf_, soil temperature (T_soil_), and SWC were recorded using a data logger (CR1000X, Campbell Scientific Instruments, Logan, UT, USA).

**Figure 6:**
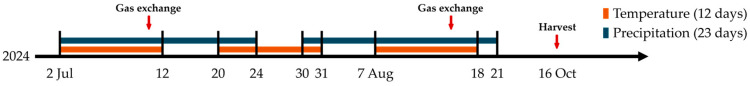
Experimental scheme of extreme climate manipulation. Orange and blue colors indicate the temperature and precipitation treatment, respectively.

The extreme climate scenarios simulated an extreme summer climate in South Korea, based on meteorological data from July and August in Seoul, from 1908 to 2023. Meteorological data for Pocheon were only available from 1997 onwards; therefore, data from Seoul, located approximately 28 km from Pocheon, were used as a substitute. T3 and T5 were defined as the 85th percentile (TX85p) and 95th percentile (TX95p) of daily maximum temperature during the referenced period, respectively. The duration of warming was determined to be 12 days, based on the number of consecutive days with temperatures exceeding TX85p and TX95p. Drought was defined as a 23-day period during which daily precipitation remained below 1 mm, based on SPEI calculated using the Hargreaves method [68]. The extreme climate scenarios are as follows (Figure 6): temperature treatment (T1st: 2–12 July; T2nd: 20–31 July; T3rd: 7–18 August), precipitation treatment (P1st: 2–24 July; P2nd: 30 July–21 August).

### 4.3. Seedling Responses

Seedling physiology, growth, and biomass responses were analyzed. Gas exchange parameters, including net photosynthesis rate (*P_n_*), transpiration rate (*E*), and stomatal conductance (*g_s_*; stomatal conductance to water vapor), were determined using a portable photosynthesis system (LI-6800, Li-Cor Inc., Lincoln, NE, USA) connected to a 2 cm × 3 cm chamber (6800-12A, Li-Cor Inc., Lincoln, NE, USA). Measurements were taken under the following conditions: a photosynthetic photon flux density of 1000 μmol m^−2^ s^−1^, a CO_2_ concentration of 420 µmol mol^−1^, ambient temperature, and 60% relative humidity. Three seedlings were randomly selected from each plot, and one leaf per seedling was measured. Gas exchange was measured on two separate dates (early: 12 July; late: 16 August) between 09:00 and 13:00 on rain-free days. Intrinsic water-use efficiency (iWUE) was calculated as the ratio of *P_n_* to *g_s_* (*P_n_*/*g_s_)*.

Seedling growth was assessed pre- and post-treatment (1 July and 6 September, respectively). Height and root collar diameter (RCD) were measured for 35 centrally located seedlings per plot to avoid edge effects, using a folding ruler and digital caliper. The absolute growth rate (AGR) was calculated as the difference between pre- and post-treatment measurements, and the relative growth rate (RGR) was calculated as AGR divided by the pre-treatment value, multiplied by 100. On 16 October, six to seven seedlings per plot, including those used for physiological measurements, were harvested to assess final biomass responses following the treatment period. Stem, leaf, and root were carefully harvested using a shovel with consistent force to minimize root loss. Samples were oven-dried at 65 °C to constant mass. Biomass was determined for roots and shoots (stem + leaf), and the root-to-shoot (R/S) ratio was calculated as the ratio of root-to-shoot biomass.

### 4.4. Data Analysis

Linear mixed-effects models were fitted using the “lmer” function in the *lme4* package to assess the effects of extreme temperature and drought manipulations on environmental variables, gas exchange (*P_n_*, *E*, *g_s_*), iWUE, and growth parameters (height, RCD, and biomass). The experiment followed a randomized complete block design, with block included as a random effect and temperature and precipitation as fixed effects. The model was specified as: Y_ijk_ = β_0_ + β_1_T_i_ + β_2_P_j_ + β_3_(T_i_ × P_j_) + b_k_ + ε_ijk_.

Model assumptions, including normality and homoscedasticity, were assessed based on the residuals. Post hoc comparisons were conducted using Tukey’s or Sidak’s adjustments. Pearson’s correlation was used to assess relationships among environmental, physiological, and growth variables (Figure S5). Statistical significance was set at *p* < 0.05. Effect sizes ($\eta_{p}^{²}$) and corresponding 95% confidence intervals for the effects of temperature and precipitation on all variables are provided (Table S2). All analyses were performed in R (version 4.4.0).

## 5. Conclusions

Our study evaluated the physiological and growth responses of *Q. variabilis* seedlings to open-field warming and drought treatments. Photosynthesis was transiently constrained in the early phase but showed signs of acclimation over time, potentially indicating a partial decoupling from stomatal conductance under elevated temperature and drought conditions. These results suggest that *Q. variabilis* possesses physiological plasticity that may enable short-term acclimation to combined stress. Notably, although warming enhanced both leaf and root biomass, the R/S ratio remained unchanged, indicating a relatively conservative carbon allocation between above- and belowground organs under stress conditions. However, given the limitations of this seedling-stage experiment and its short duration, caution is required in extrapolating these findings. While *Q. variabilis* appears to maintain physiological flexibility and stable biomass allocation under short-term warming and drought, its long-term responses to prolonged or repeated stress remain uncertain. Further studies across extended time scales, multiple species, and different developmental stages are needed to better understand its adaptive capacity under future climate scenarios.

## Figures and Tables

**Table 1:** *F*–values for the effects of precipitation, temperature, and their interaction on the environmental factors (two-way ANOVA).

Variables	T_leaf_			T_soil_			SWC	
	T1st	T2nd	T3rd	T1st	T2nd	T3rd	P1st	P2nd
Prec	1.24	0.08	2.08	1.48	0.01	2.51	4.84 *	1.21
Temp	431.34 ***	15.12 ***	183.67 ***	60.30 ***	16.64 ***	27.52 ***	0.16	0.30
Prec × Temp	0.09	0.22	1.47	0.56	1.58	0.87	0.05	0.13

Prec, precipitation; Temp, temperature; T_leaf_, leaf temperature; T_soil_, soil temperature; SWC, soil water content. Temperature treatments: T1st (2–12 July), T2nd (19–30 July), T3rd (7–18 August). Precipitation treatments: P1st (2–24 July), P2nd (30 July–21 August). Asterisks indicate significant differences (* *p* < 0.05; *** *p* < 0.001).

**Table 2:** *F*–values for the effects of precipitation, temperature, and their interaction on physiology and growth (two-way ANOVA).

Variables	*P_n_*		*E*		*g_s_*		iWUE	
	Early	Late	Early	Late	Early	Late	Early	Late
Prec	5.10 *	0.05	1.61	0.12	0.50	0.05	3.18	1.62
Temp	0.85	1.20	2.08	2.72	2.34	3.34	2.52	6.26 *
Prec × Temp	3.81	0.81	1.20	0.85	5.48 *	0.54	0.18	0.18
	**RGR_Height_**	**RGR_RCD_**	**Stem**	**Leaf**	**Shoot**	**Root**	**R/S ratio**	
Prec	0.04	7.41 *	0.01	0.02	0.01	0.47	0.07	
Temp	0.63	2.90	3.24	4.76 *	4.31 *	4.60 *	1.37	
Prec × Temp	1.40	2.06	0.27	0.63	0.48	1.19	1.06	

Prec, precipitation; Temp, temperature; *P_n_*, net photosynthetic rate; *E*, transpiration rate; *g_s_*, stomatal conductance; iWUE, intrinsic water-use efficiency; RGR_Height_, relative growth rate of height; RGR_RCD_, relative growth rate of root collar diameter; Stem, stem biomass; Leaf, leaf biomass; Shoot, shoot biomass (stem + leaf biomass); Root, root biomass; R/S ratio, root-to-shoot ratio. Early and late phases indicate measurement time points during the treatment period (12 July and 16 August, respectively). Asterisks indicate significant differences (* *p* < 0.05).

## Data Availability

The data presented in this study are available on request from the corresponding author.

## Supplementary Materials

The following supporting information can be downloaded at: https://www.mdpi.com/article/10.3390/plants15091354/s1, Table S1: *F*-values for the effects of precipitation, temperature, and their interaction on the absolute growth rates of height and RCD (two-way ANOVA); Table S2. Effect sizes ($\eta_{p}^{²}$) and 95% confidence intervals for the effects of extreme warming and drought conditions.; Figure S1: Changes in environmental factors under temperature and precipitation manipulation. (a) Leaf temperature; (b) soil temperature; (c) soil water content and daily precipitation. Bars indicate daily precipitation. TC: ambient temperature; T3: +3 °C warming; T5: +5 °C warming; PC: ambient precipitation; DR: drought condition. Asterisks indicate significant differences (* *p* < 0.05, *** *p* < 0.001), while n.s. indicates non-significance (*p* > 0.05).; Figure S2: Changes in growth under extreme warming and drought conditions. (a) Absolute growth rate (AGR) of height; (b) AGR of root collar diameter (RCD). TC: ambient temperature; T3: +3 °C warming; T5: +5 °C warming; PC: ambient precipitation; DR: drought condition. Asterisks denote statistically significant differences (* *p* < 0.05, ** *p* < 0.01). Different letters indicate significant differences between precipitation treatments within each temperature.; Figure S3: Changes in stem and leaf biomass under warming and drought conditions. (a) Stem biomass; (b) leaf biomass. TC: ambient temperature; T3: +3 °C warming; T5: +5 °C warming; PC: ambient precipitation; DR: drought condition. Asterisks denote statistically significant differences (* *p* < 0.05). Different letters indicate significant differences among temperature treatments.; Figure S4: Relationship between net photosynthesis rate (*P_n_*) and stomatal conductance (*g_s_*). Early and late phases indicate measurement time points during the treatment period (12 July and 16 August, respectively). TC: ambient temperature; T3: +3 °C warming; T5: +5 °C warming. Point color indicates leaf temperature (T_leaf_). Solid, dotted, and dashed lines represent TC, T3, and T5, respectively. Asterisks indicate significant interactions between *g_s_* and temperature treatment within each phase.; Figure S5: Correlations among environmental factors, physiological traits, and growth (biomass) parameters. Values represent Pearson’s correlation coefficients. Asterisks indicate significance levels (* *p* < 0.05; ** *p* < 0.01; *** *p* < 0.001), and numbers correspond to correlation coefficients.
