# Supplementary material for: Effects of Combined Extreme Warming and Drought on the Physiology and Growth of Quercus variabilis Blume Seedlings
Source: Plants (Basel). 2026 Apr 28;15(9):1354. doi: 10.3390/plants15091354 (PMC13165138; doi:10.3390/plants15091354)
Supplement: Supplementary file 1 [file plants-15-01354-s001.zip › plants-4247318-supplementary.pdf]

plants

MDPI

Supplementary material

Supplementary Table S1. *F*-values for the effects of precipitation, temperature, and their interaction on the absolute growth rates of height and RCD (two-way ANOVA)

Variables	AGR_{Height}	AGR_{RCD}
Prec	0.12	7.80*
Temp	1.52	2.70
Prec × Temp	2.12	2.27

Prec, precipitation; Temp, temperature; AGR_{Height}, absolute growth rate of height; AGR_{RCD}, absolute growth rate of root collar diameter. Asterisks indicate significant differences (* $p < 0.05$).

Supplementary Table S2. Effect sizes (η_p^2) and 95% confidence intervals for the effects of extreme warming and drought conditions.

Intercept		Prec	Temp	Prec×Temp	TCDR		TCPC		T3DR		T3PC		T5DR		T5PC	
		η_p^2	η_p^2	η_p^2	2.5%	97.5%	2.5%	97.5%	2.5%	97.5%	2.5%	97.5%	2.5%	97.5%	2.5%	97.5%
**Parameter**																
**Early**	P_n	0.338	0.146	0.432	6.339	9.45	7.626	10.737	6.119	9.230	9.421	12.532	8.127	11.238	7.488	10.599
	E	0.139	0.294	0.194	1.536	2.848	2.138	3.450	1.805	3.117	2.365	3.677	2.561	3.873	2.335	3.646
	g_s	0.048	0.319	0.523	92.617	155.065	103.733	166.181	105.091	167.539	131.461	193.909	159.901	222.349	97.513	159.961
	**iWUE**	0.241	0.335	0.035	56.734	77.531	62.201	82.998	47.384	68.181	52.517	73.314	45.337	66.134	55.585	76.382
**Late**	P_n	0.005	0.193	0.139	4.914	10.312	4.851	10.249	4.829	10.227	6.696	12.094	7.339	12.737	6.165	11.563
	E	0.012	0.352	0.146	2.006	4.582	2.809	5.385	2.980	5.555	3.377	5.953	4.128	6.703	3.437	6.013
	g_s	0.005	0.401	0.098	78.173	227.291	110.563	259.682	156.405	305.523	132.195	281.314	188.177	337.296	162.987	312.105
	**iWUE**	0.14	0.556	0.034	48.698	66.688	41.577	59.567	37.086	55.077	33.974	51.965	32.130	50.121	29.477	47.467
**RGR of height**		0.004	0.112	0.219	1.789	3.857	0.841	2.909	1.001	3.069	1.607	3.675	0.788	2.857	0.905	2.973
**RGR of RCD**		0.426	0.367	0.292	0.043	17.589	0.919	18.465	0.279	17.825	4.389	21.934	0.861	18.406	10.585	28.131
**AGR of height**		0.012	0.233	0.298	0.489	0.908	0.252	0.672	0.272	0.691	0.43	0.849	0.218	0.638	0.214	0.634
**AGR of height**		0.438	0.351	0.312	0.019	0.532	0.043	0.555	0.003	0.515	0.104	0.617	0.024	0.537	0.281	0.793
**Stem biomass**		0.001	0.393	0.051	0.801	1.13	0.824	1.153	0.739	1.069	0.671	1.000	0.881	1.211	0.913	1.242
**Leaf biomass**		0.002	0.488	0.111	1.503	2.207	1.671	2.375	1.512	2.215	1.361	2.065	1.852	2.556	1.890	2.594
**Shoot biomass**		0.001	0.463	0.088	2.339	3.302	2.531	3.493	2.286	3.249	2.068	3.030	2.769	3.732	2.838	3.801
**Root biomass**		0.045	0.479	0.192	5.839	7.795	6.285	8.241	6.925	8.881	6.653	8.609	7.859	9.815	6.944	8.899
**R/S ratio**		0.007	0.216	0.175	2.199	2.973	2.409	3.183	2.511	3.286	2.652	3.426	2.582	3.356	2.338	3.112

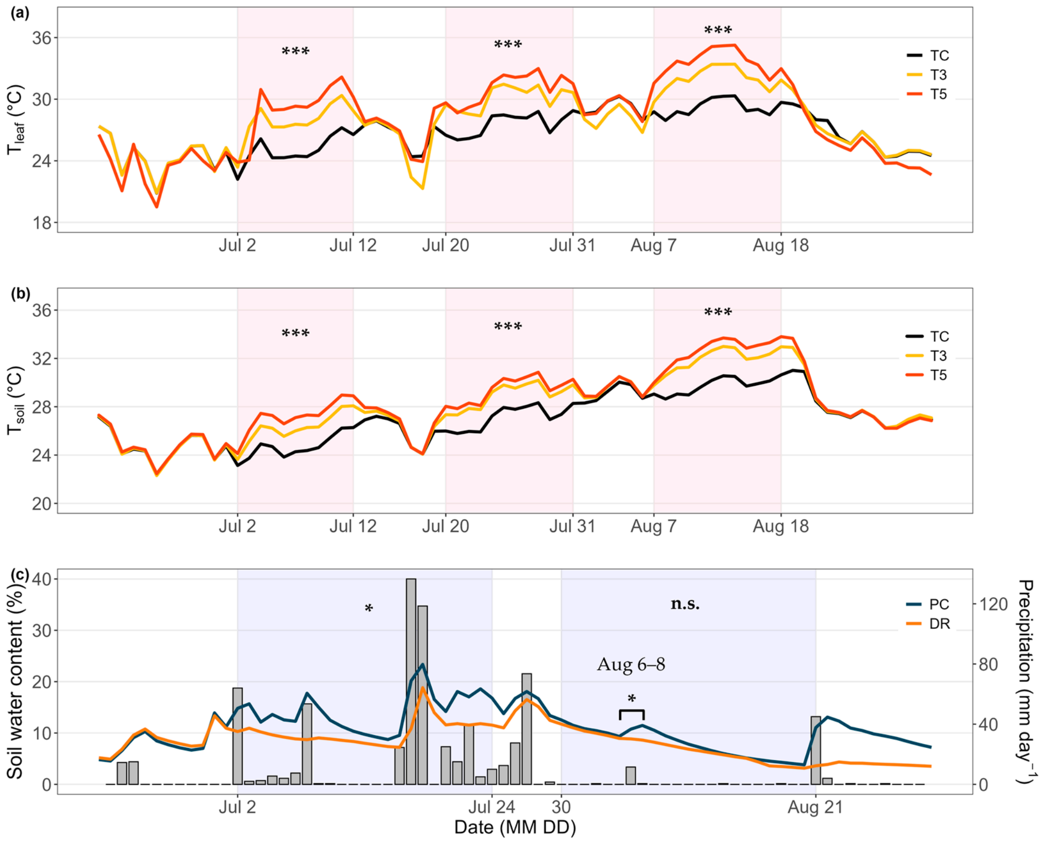

Supplementary Figure S1. Changes in environmental factors under temperature and precipitation manipulation. (**a**) Leaf temperature (T_{leaf}); (**b**) soil temperature (T_{soil}); (**c**) soil water content and daily precipitation. Bars indicate daily precipitation. TC: ambient temperature; T3: +3 °C warming; T5: +5 °C warming; PC: ambient precipitation; DR: drought condition. Asterisks indicate significant differences (* $p < 0.05$, *** $p < 0.001$), while n.s. indicates non-significance.

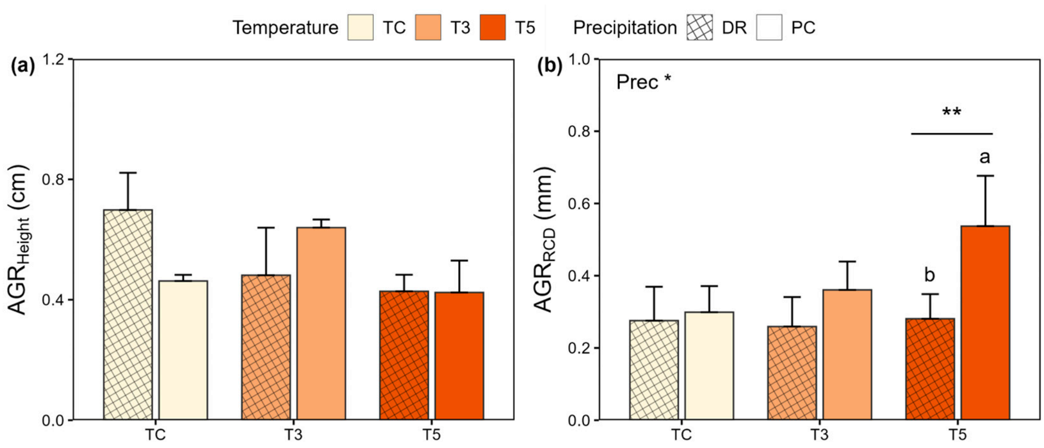

Supplementary Figure S2. Changes in growth under extreme warming and drought conditions. (**a**) Absolute growth rate (AGR) of height; (**b**) AGR of root collar diameter (RCD). TC: ambient temperature; T3: + 3 °C warming; T5: + 5 °C warming; PC: ambient precipitation; DR: drought condition. Asterisks denote statistically significant differences (* $p < 0.05$, ** $p < 0.01$). Different letters indicate significant differences between precipitation treatments within each temperature.

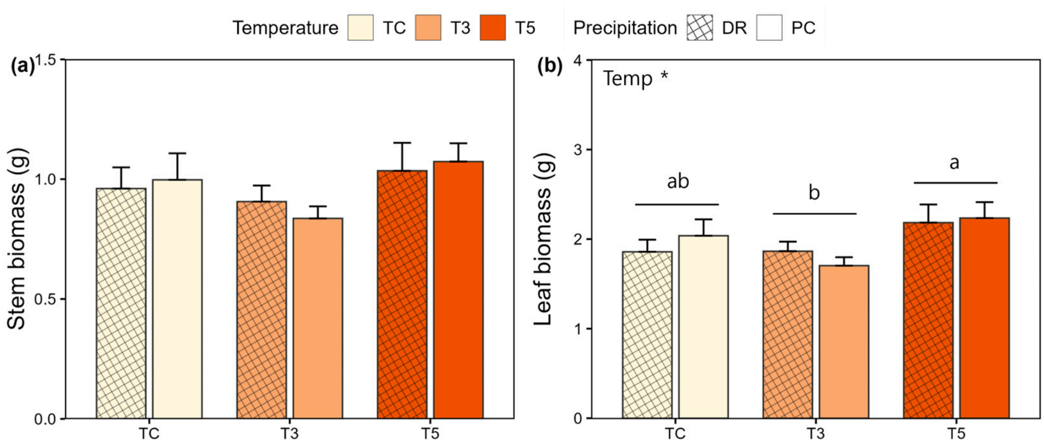

Supplementary Figure S3. Changes in stem and leaf biomass under warming and drought conditions. (**a**) Stem biomass; (**b**) leaf biomass. TC: ambient temperature; T3: + 3 °C warming; T5: + 5 °C warming; PC: ambient precipitation; DR: drought condition. Asterisks denote statistically significant differences (* $p < 0.05$). Different letters indicate significant differences among temperature treatments.

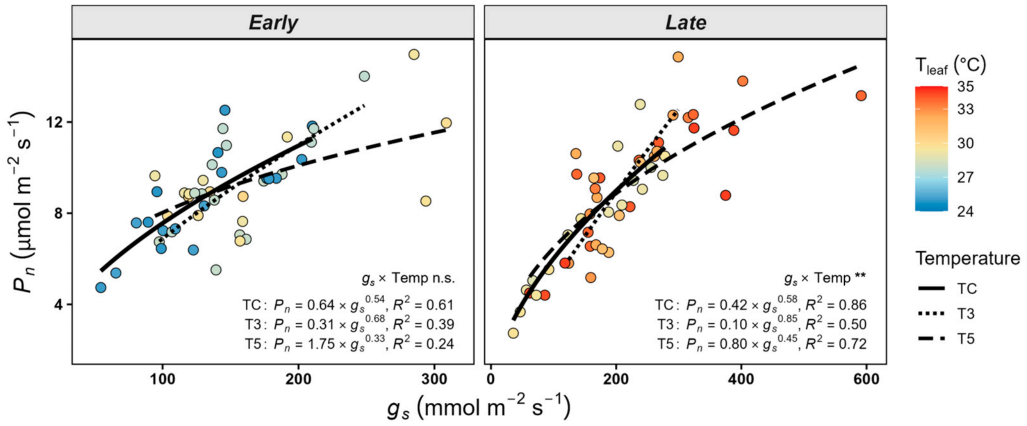

Supplementary Figure S4. Relationship between net photosynthesis rate (P_n) and stomatal conductance (g_s). Early and late phases indicate measurement time points during the treatment period (12 July and 16 August, respectively). TC: ambient temperature; T3: + 3 °C warming; T5: + 5 °C warming. Point color indicates leaf temperature (T_{leaf}). Solid, dotted, and dashed lines represent TC, T3, and T5, respectively. Asterisks indicate significant interactions between g_s and temperature treatment within each phase (** $p < 0.01$).

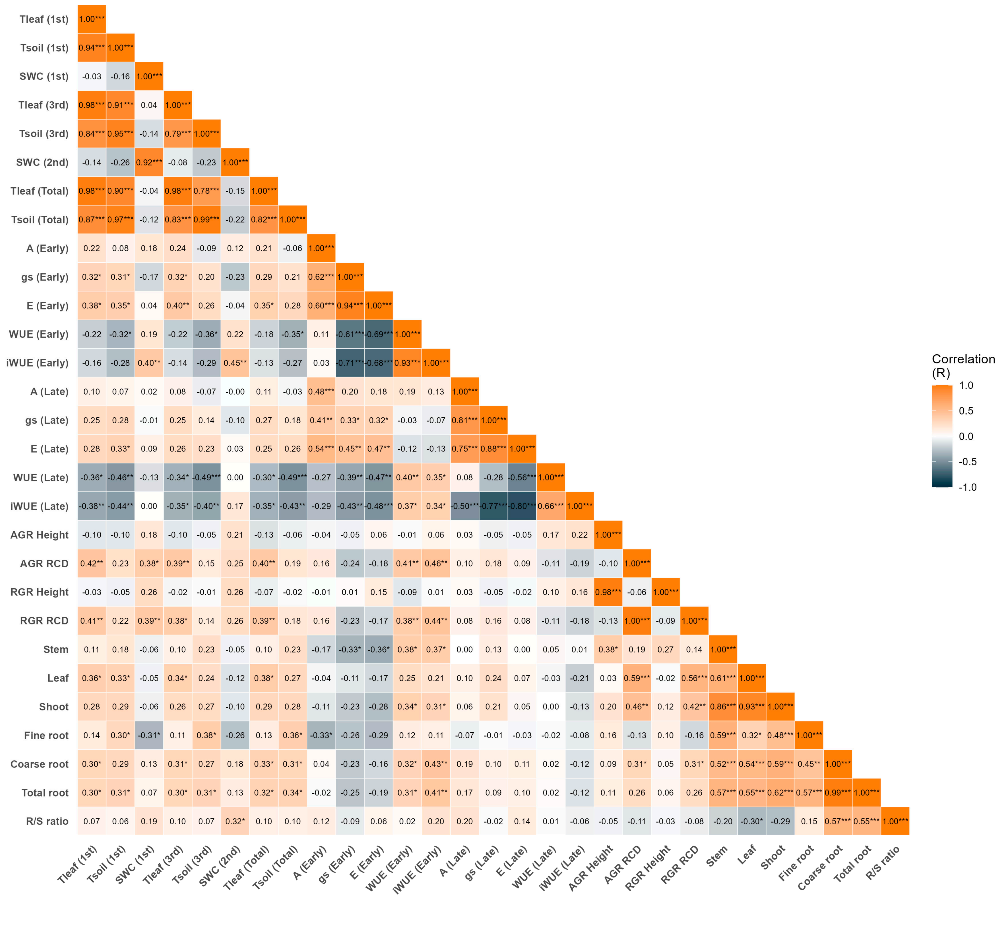

Supplementary Figure S5. Correlations among environmental factors, physiological traits, and growth (biomass) parameters. Values represent Pearson's correlation coefficients. Asterisks indicate significance levels (* $p < 0.05$; ** $p < 0.01$; *** $p < 0.001$), and numbers correspond to correlation coefficients.
